# A novel homozygous splice site variant in *ARL2BP* causes a syndromic autosomal recessive rod-cone dystrophy with situs inversus, asthenozoospermia, unilateral renal agenesis and microcysts

**DOI:** 10.1186/s12920-024-01868-w

**Published:** 2024-04-22

**Authors:** Giorgio Placidi, Elena D’Agostino, Paolo Enrico Maltese, Maria Cristina Savastano, Gloria Gambini, Stanislao Rizzo, Gabriele Bonetti, Matteo Bertelli, Pietro Chiurazzi, Benedetto Falsini

**Affiliations:** 1 UOC Oculistica, Fondazione Policlinico Universitario “A. Gemelli” IRCCS, Largo Gemelli 8, 00168 Rome, Italy; 2 MAGI’S LAB, 38068 Rovereto, Italy; 3 MAGI EUREGIO, 39100 Bolzano, Italy; 4 Istituto di Oftalmologia, Università Cattolica del Sacro Cuore, Largo Francesco Vito 1, 00168 Rome, Italy; 5 Istituto di Neuroscienze, Consiglio Nazionale Delle Ricerche, Pisa, Italy; 6 MAGISNAT, Atlanta Tech Park, 107 Technology Parkway, 30092 Peachtree Corners, GA USA; 7 Istituto di Medicina Genomica, Università Cattolica del Sacro Cuore, Largo Francesco Vito 1, 00168 Rome, Italy; 8 UOC Genetica Medica, Fondazione Policlinico Universitario “A. Gemelli” IRCCS, Largo Gemelli 8, 00168 Rome, Italy

**Keywords:** ARL2BP, Syndromic rod-cone dystrophy, Renal agenesis, Cryptorchidism

## Abstract

**Background:**

This report presents a clinical case of syndromic rod-cone dystrophy due to a splice site variant in the *ARL2BP* gene causing situs inversus, asthenozoospermia, unilateral renal agenesis and microcysts. The presence of renal agenesis and cryptorchidism expands the clinical manifestations due to *ARL2BP* variants. The detailed, long-term follow-up contributes valuable insights into disease progression, aiding clinical diagnosis and patient management.

**Case Presentation:**

The male patient complained of photophobia as the first symptom when he was 20 years old followed by nyctalopia, loss of central visual acuity and peripheral visual field ten years later. Genetic analysis identified a likely pathogenic homozygous variant (c.294-1G > C) involving the splicing acceptor site of intron 4. Reported symptoms together with full-field stimulus threshold testing, electroretinogram and advanced multimodal imaging allowed us to recognize the typical characteristics of a mixed retinal dystrophy. Despite the end-stage retinal disease, this patient still retained a useful residual vision at 63 years and had a slow disease progression during the last 5 years of evaluation.

**Discussion and conclusions:**

Our findings underscore the variable clinical presentation of *ARL2BP* variants, emphasizing the importance of a nuanced approach in diagnosing and managing patients. The presence of renal cysts warrants consideration of a differential diagnosis, particularly with Senior-Loken (SLS), Bardet-Biedl (BBS) and Joubert syndromes (JS) but also with Short Rib Thoracic Dysplasia 9, highlighting the need for careful phenotypic evaluation in these cases.

## Background

Inherited retinal dystrophies (IRDs) are a large group of clinically and genetically heterogeneous diseases known to cause severe visual impairment and blindness. Some of them, called ciliopathies, can affect multiple proteins acting at the level of the cilium–centrosome complex and involve different anatomical districts. Several syndromic IRDs such as Joubert, Bardet–Biedl and the Senior-Loken syndromes have already been classified as ciliopathies.

Among the genes potentially causative of ciliopathies when altered, biallelic pathogenic variants in *ARL2BP* (OMIM *615,407) can cause autosomal recessive Retinitis Pigmentosa (arRP) with or without situs inversus (OMIM #615,434). *ARL2BP* encodes the ADP-ribosylation factor-like 2 binding protein, a centrosome-associated protein linked to ARL2 and ARL3, regulating the assembly of multi-protein complexes [1].

ADP-ribosylation factor-like 2 binding protein is localized at the distal side of connecting cilia and in the periciliary extension of the inner segment, where the intraflagellar transport (IFT) is highly active [2]. IFT is responsible for the bidirectional movement of proteins and vesicles between the base and the ciliary tip. Given its role, it is conceivable that *ARL2BP* variants result in an altered displacement of proteins and factors moving between the outer and inner segments of the photoreceptors.

When the protein is dysfunctional, it also causes cilia shortening [1].

In addition, Moye et al. [3] demonstrated the correlation between *ARL2BP* pathogenic variants and malformations of the photoreceptor outer segments (Oss) in mouse models. Based on their analysis, this gene would be necessary for photoreceptor ciliary doublet formation and axoneme elongation. Hence, a potential role in disk neogenesis in the outer segments has been suggested for this gene [1].

Previous studies reported patients suffering from retinitis pigmentosa with different systemic signs due to ciliary dysfunction [4–6].

In this report, we present a patient affected by a novel homozygous splicing variant in the *ARL2BP* gene. The phenotypic manifestations determined by his splice variant show that many signs fragmentarily described in association with this condition both in humans and in mouse models are present in this subject. The presence of renal agenesis and cryptorchidism expands the clinical manifestations due to *ARL2BP* variants.

## Case presentation

In June 2018, a 58 years old patient with a previous diagnosis of retinitis pigmentosa was evaluated at the Center for Inherited Retinal Degenerations of Fondazione Policlinico Universitario “A. Gemelli” IRCCS.

The patient presented situs inversus for the spleen and liver, located in the right and left hypochondrium, respectively. The stomach was displaced to the right and the vena cava to the left.

Abdominal ultrasound showed unilateral renal agenesis concerning the left kidney while the right one was *in situ* and appeared enlarged with a microcyst. Hypertension was present. The prostate was increased in size and had a heterogeneous structure and a millimetric cystic formation. Cardiac examination with transthoracic echocardiogram documented mitral valve prolapse without regurgitation. In a previous urological visit, asthenozoospermia and left cryptorchidism were reported so that the patient underwent orchiectomy in 1996 at the age of 31.

The patient reported a history of night blindness (NB) and visual field constriction (VFC) since he was 30 years old, preceded by photophobia and episodes of photopsia already noted ten years before. Diagnosis of arRP was made when he was 32 years old and both NB and VFC became prominent at 35, when light aversion also developed. The patient could no longer drive by the age of 39.

When he was 49 years old, visual acuity was 20/200 in both eyes with a fundus characterized by attenuated vessels, rare bone spicule-like deposits scattered in mid-periphery and prominent patches of 360° chorioretinal nummular atrophy in far periphery. Goldmann Visual Field showed a marked constriction in both eyes (< 10° with the V/4e target) while optical coherence tomography (OCT) showed a widespread reduction of the outer nuclear layer and RNFL attenuation with RGC axonal loss, more evident in the right eye (RE) compared to the left (LE).

At 50 years a sudden drop in visual acuity occurred (BCVA RE: hand motion; BCVA LE: 20/400) with coincidental findings of an active anterior uveitis treated with periocular injections of depot steroids in both eyes. The patient underwent a Western Blot anti-retinal autoantibodies against 30-kDa, 35-kDa and 44-kDa proteins using targeted serological analyses and tested positive. He was negative for anti-optic nerve autoantibodies.

Since the patient represented a sporadic case of syndromic RP in his family and reported being the son of first cousins, we considered the autoimmune reaction a secondary phenomenon to a primary form of true retinitis pigmentosa.

During our first ophthalmological evaluation (at the age of 58), BCVA was hand motion in RE and 20/125 (0.8 LogMAR) in LE. Full-field electroretinogram (ERG) according to the standard ISCEV showed scotopic and photopic responses not differentiable from background noise in both eyes. Responses to visual evoked potentials (VEPs) were markedly reduced. Fundus oculi examination confirmed the signs already described.

After the visit, the patient underwent genetic testing by Next-Generation Sequencing (NGS). The methods of genetic analysis and the list of analyzed genes are described elsewhere [7]. In brief, NGS was conducted using a MiSeq personal sequencer, which considered a custom-designed gene panel analyzed by an in-house developed pipeline [8].

Genetic testing identified the homozygous variant c.294-1G > C in *ARL2BP* gene (Table 1). The variant has never been described before as a disease-related variant, while it is reported in 2 heterozygotes in the gnomAD population database; however, according to the ACMG criteria [9], it is considered likely pathogenic because it disrupts the splice acceptor site upstream of exon 5. Indeed, it is predicted (https://franklin.genoox.com/; https://varsome.com/; https://autopvs1.bgi.com/; last accessed 23 october 2023) to remove 19.7% of transcript through exon skipping, thus likely leading to nonsense-mediated mRNA decay in a gene where loss-of-function is a known mechanism of disease [1, 5]. No other variants that could potentially explain or modify the phenotype were detected by genetic testing (Table 1).

It was not possible to perform a segregation test since both parents were deceased and the patient is an only child. However the reported consanguinity and homozygosity of the variant allow the assumption that the variants are *in trans*.

**Table 1:** Genetic variants identified in the study subject

Gene	OMIM gene	Nucleotide change	Amino acid change	Allelic status	rsID	Classification
*ARL2BP*	615,407	NM_012106.4:c.294-1G > C	p.(?)	Hom	rs1567526361	LP (PVS1, PM2)
*IFT172*	607,386	NM_015662.2:c.4933G > A	NP_056477.1:p.(Val1645Ile)	Het	rs149117098	B
*EYS*	612,424	NM_001142800.1:c.3586T > C	NP_001136272.1:p.(Cys1196Arg)	Het	rs374409854	B
*FLVCR1*	609,144	NM_014053.3:c.1028T > C	NP_054772.1:p.(Ile343Thr)	Het	rs774455543	VUS

*Legend* Gene name, OMIM gene number, nucleotide and amino acid change, allelic status, dbSNP Reference SNP cluster ID (rsID) number and ACMG classification are reported. LP = Likely Pathogenic; VUS = Variant of Uncertain Significance; B = Benign; PVS1 = Very Strong Pathogenic criteria; PM2 = Moderate Pathogenic criteria: Hom = Homozygous; Het = Heterozygous; B = Benign

The latest assessment of the patient was performed in October 2023, when he was 63 years old.

**Fig. 1:**
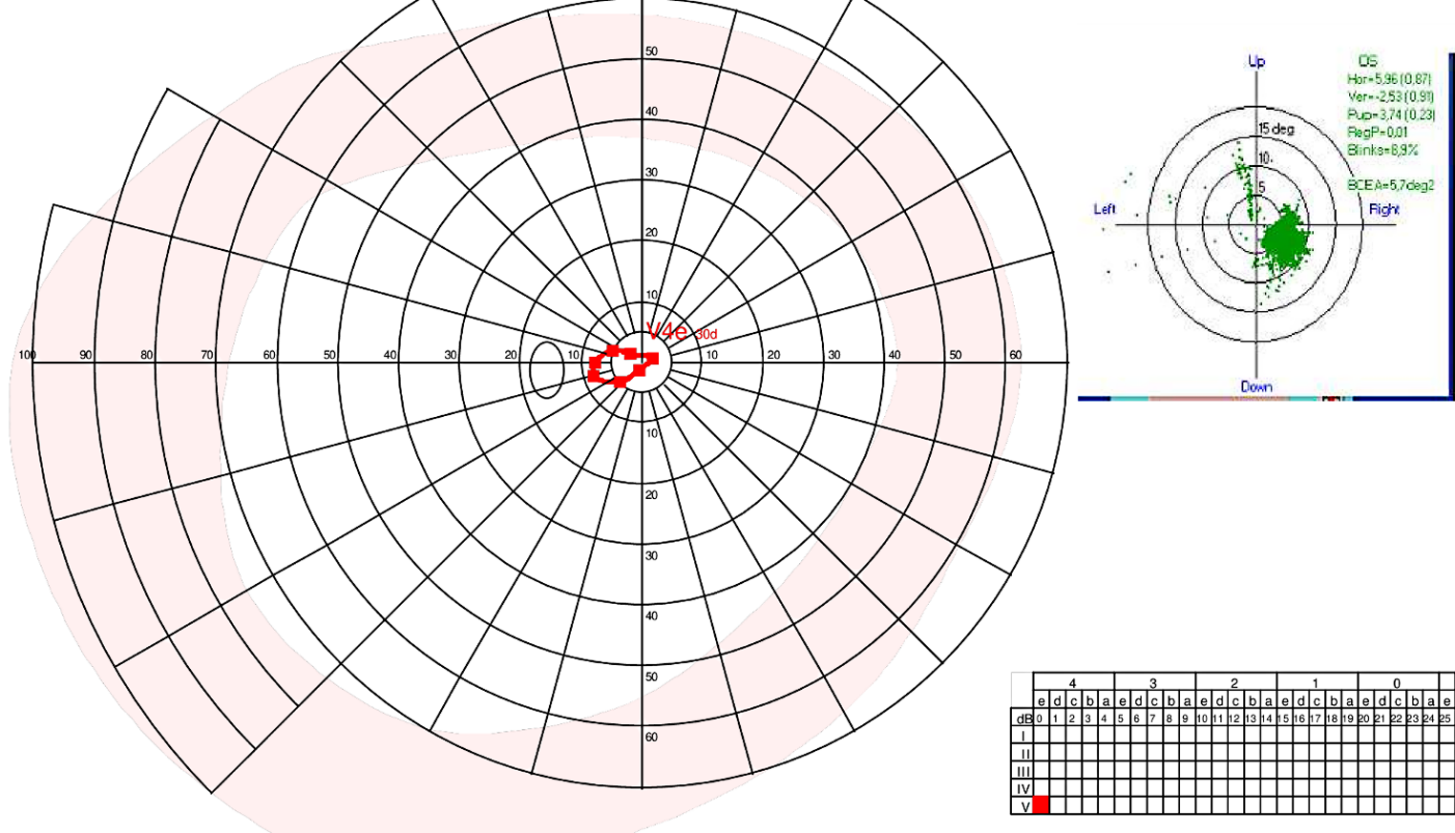
Visual field extension with V/4e target in LE. *Legend* The patient has a stable fixation but approximately 5 degrees off-center to the bottom right. The residual visual field is slightly shifted to the temporal side. The test is reliable

In addition to the previous examinations, MonCvONE-PRO Goldmann manual perimetry, advanced multimodal imaging and full-field stimulus threshold testing (FST) [10] were performed. BCVA remained unchanged in both eyes (RE: hand motion; LE: 20/125). MonCvONE-PRO Goldmann manual perimetry was unenforceable in the RE due to lack of fixation. In the LE, the patient still retained a tubular visual field of 30 square degrees and maximum diameter of 10° with V/4e target. Eye tracking system showed a reliable and slightly decentralized fixation between 2 and 10 bottom right degrees (see Fig. 1).

**Fig. 2:**
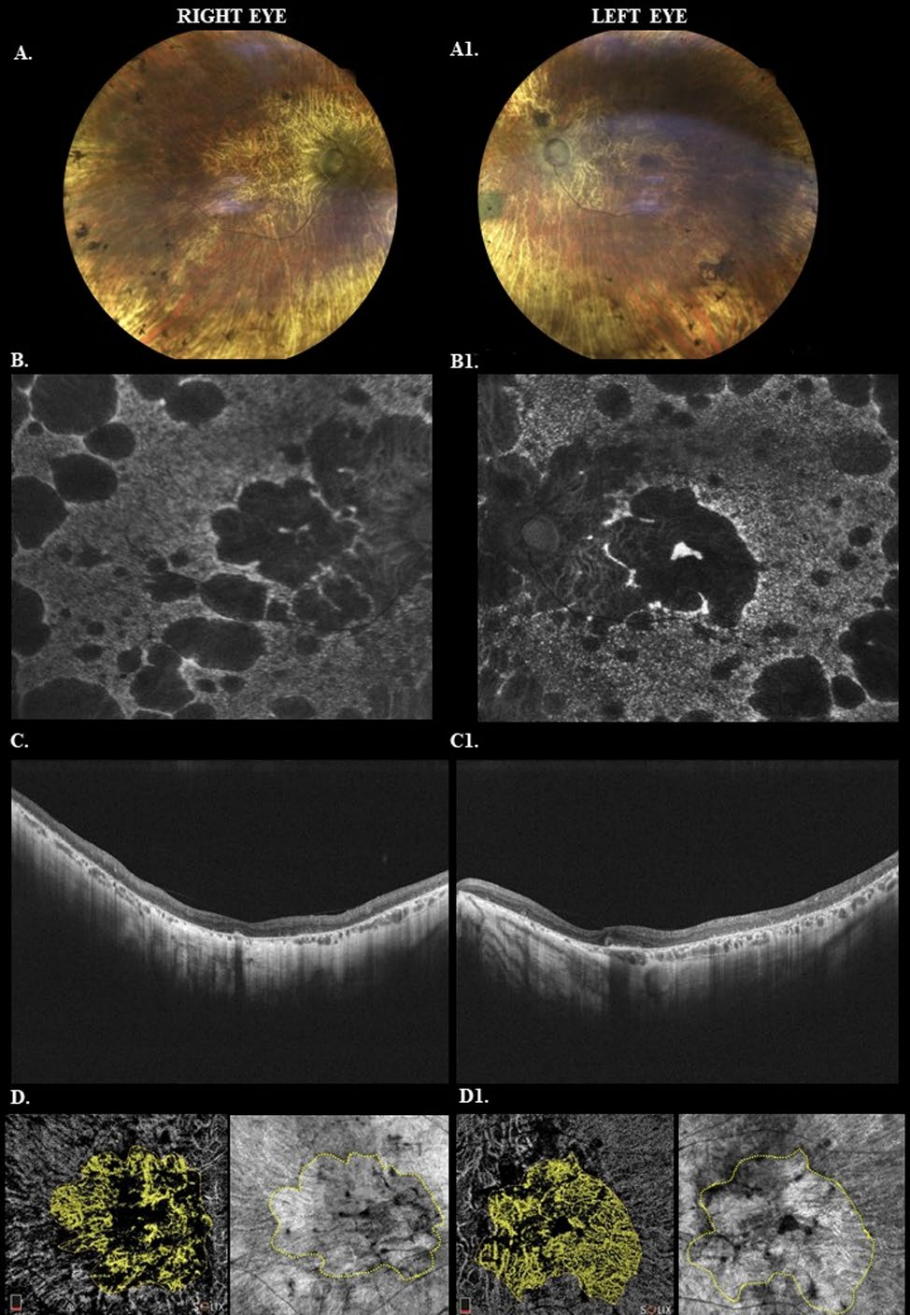
Comprehensive advanced multimodal imaging evaluation. *Legend* Color fundus, autoflurescence, OCT B-scan images in RE (**A**-**C**) and LE (**A1**-**C1**), Angio OCT and enface in right eye (**D**) and in left eye (**D1**) (**A**-**A1**). The color fundus (Confocal fundus scanner, Eidon, Centervue, Padova, Italy) images show bone spicule pigmentations and retinal atrophy in all peripheral retina and at posterior pole. Through the atrophy of the retinal pigment epithelium (RPE) transpire the large vessels of the choroid also clear at the posterior pole. In addition, the optic nerve appears waxy and the arterial vessels are threadlike and of poor visualization. (**B**-**B1**) Autofluorescence (FAF) (Scanning laser Ophthalmoscope Mirante, Nidek, Milano, Italy) images highlight the areas of rounded atrophy at posterior pole, in peripapillary region and in middle periphery. The focal area of hyperautofluorescence at the foveal level shows central sparing by partial preservation of RPE and outer retina. The perifoveael hyperautofluorescent halo represents the area of increasing atrophy delineating future enlargement of atrophy in macula area. (**C**-**C1**) OCT B-Scan (Optovue Solix Visionix, Inc. Freemont, CA, USA) shows diffuse atrophy of the inner and outer retina with disappearance of the ellipsoid zone and external limiting membrane. Severe backscattering denotes diffuse atrophy of the RPE. Shadow cone effect in subfoveal region indicates the presence of preservation of the outer retina with associated underlying junction/EPR complex. (**D**-**D1**) Angio OCT (Optovue Solix Visionix, Inc. Freemont, CA, USA) shows choroidal transparency by choriocapillary atrophy of the RPE. Enface images show the delimited area of RPE atrophy (indicated by a yellow dash line) from the area where the RPE is still preserved

The patient underwent a comprehensive advanced multimodal imaging evaluation, as shown in Fig. 2.

**Fig. 3:**
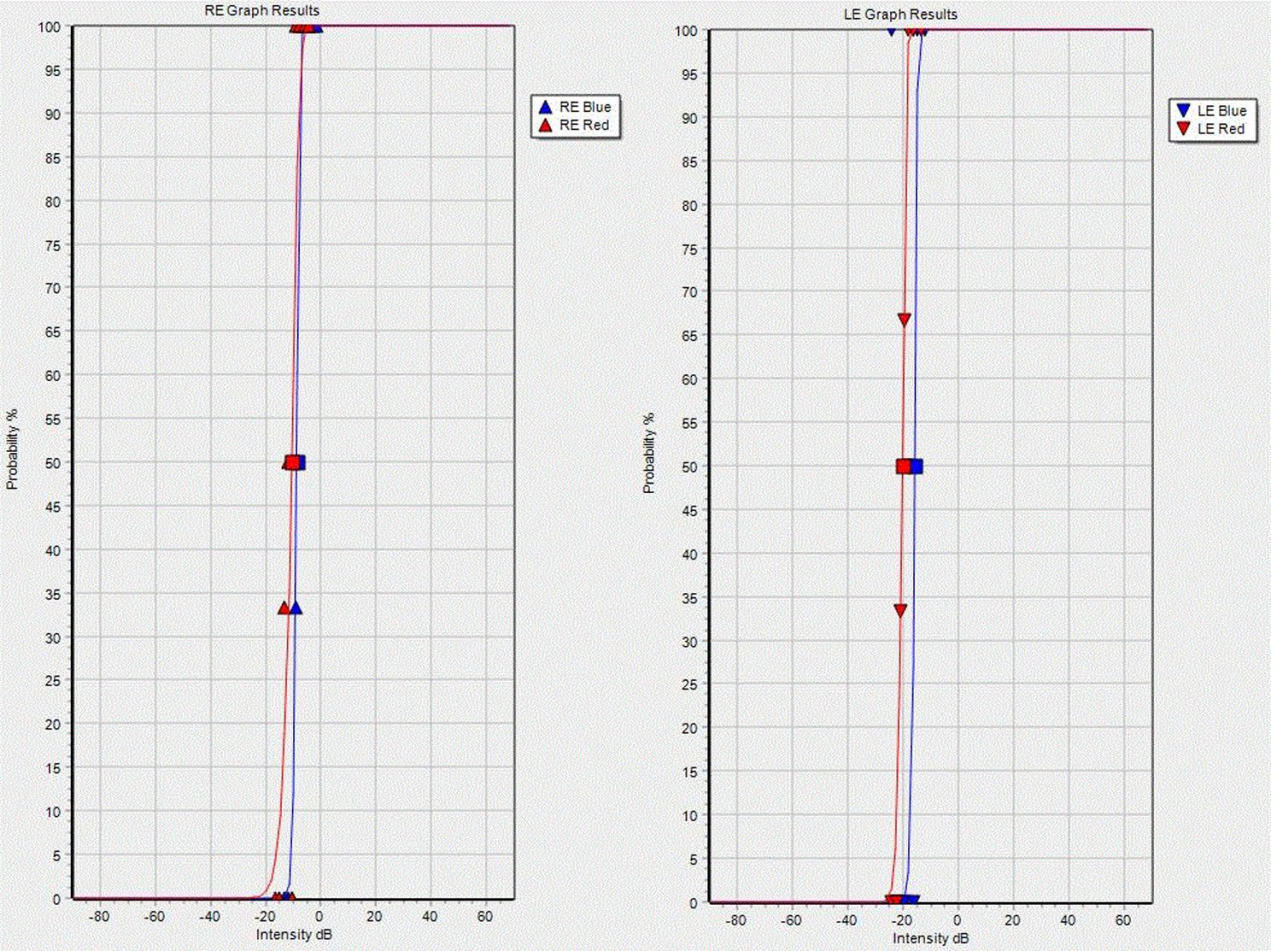
Chromatic Full-field stimulus threshold testing. *Legend* Chromatic FST show similar responses between cone and rod mediated thresholds in both eyes suggesting functional loss of rod vision and cone-mediation for all stimuli

FST examinations were performed using the Espion Color Dome^®^ (Diagnosys, Cambridge, UK). Mean FST responses were obtained from scotopic white, blue and red stimuli. FST sensitivity to white stimulus was − 0.863 log cd.s/m^2^ (-8.6 dB) on the RE and − 1.740 log cd.s/m^2^ (-17.4 dB) on the LE. FST sensitivity to blue stimulus was − 0.863 log cd.s/m^2^ (-8.6 dB) on the RE and − 1.581 log cd.s/m^2^ (-15.8 dB) on the LE. FST sensitivity to red stimulus was − 1.058 log cd.s/m^2^ (-10.5 dB) on the RE and − 2.011 log cd.s/m^2^ (-20.1 dB) on the LE (see Fig. 3).

**Fig. 4:**
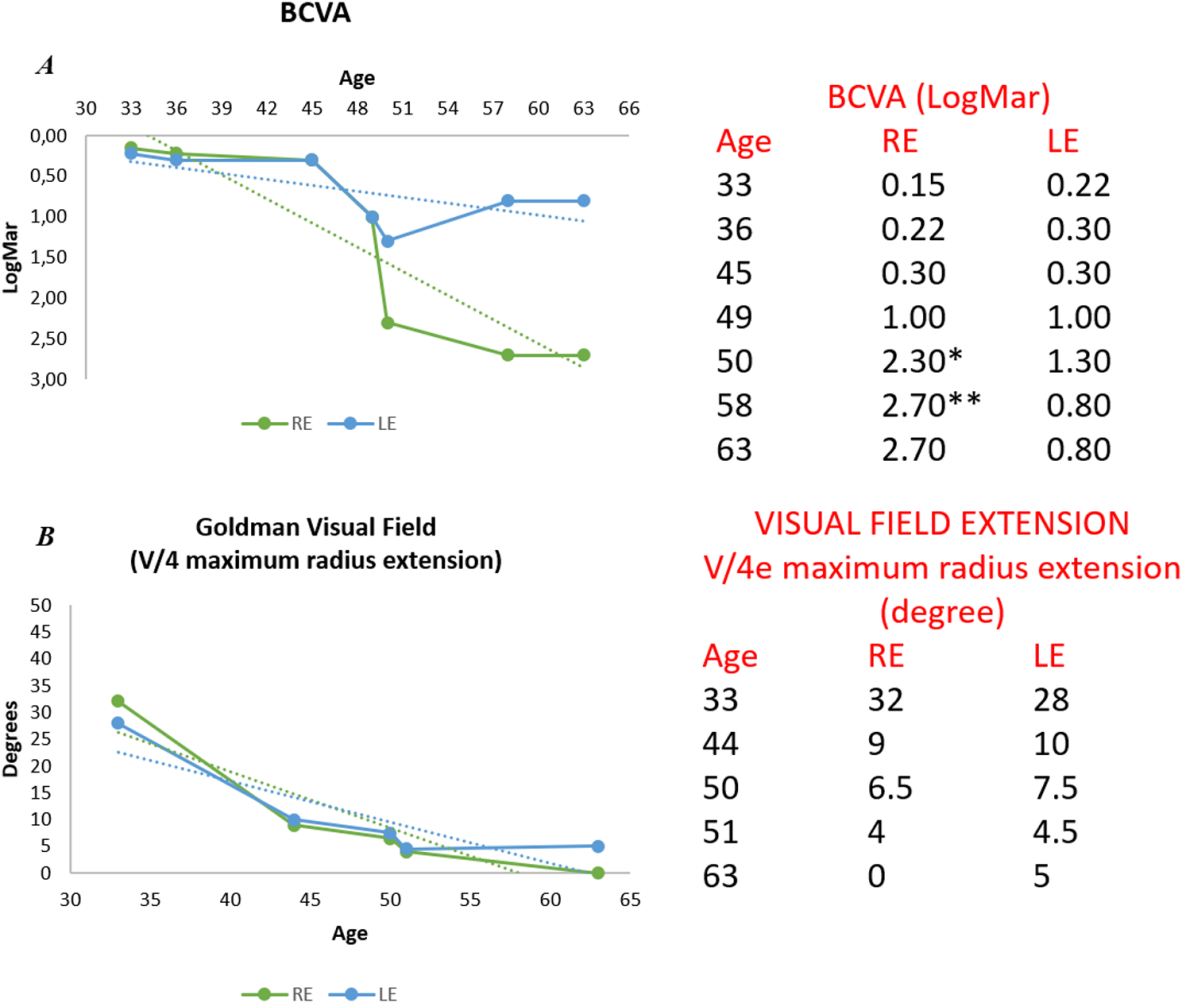
Retinal dystrophy progression over time followed by BCVA and visual field extension. *Legend* Graph (**A**) shows the progressive decline of BCVA over 30 years of disease, from diagnosis (33 years old) to the last evaluation (63 years old). Graph (**B**) highlights the progressive loss of visual field, expressed as maximum radius extension in both eyes. The numerical data are reported in the corresponding tables on the right. * 2.30 LogMar corresponds to Hand Motion. **2.70 LogMar corresponds to Light Perception

Previous clinical data collection concerning this patient allowed us to analyze the rate of progression of his retinal dystrophy over time. All available data relating to BCVA and visual field extension are shown in Fig. 4.

Ever since the patient was 36 years old, ERGs were extinct in both eyes. The latest performed in our clinic date back to 2018 and 2023, when the patient was 58 and 63, respectively. In both measurements, the responses were poorly differentiable from the background noise.

However, despite the severe vision loss, the remaining visual field and the BCVA in the LE still represent for this patient a functional residual vision allowing him to use video magnifiers or other visual aids. With an appropriate zoom, the patient was still able to read and write text messages on his mobile phone. Furthermore, BCVA in his better eye was the same in comparison with 5 years before. This evidence indicates a slow progression of the disease.

## Discussion and conclusions

The description of cases concerning patients with biallelic likely pathogenic variant in the *ARL2BP* gene are quite rare in literature. The high intra- and inter-subject variability is a common feature of all the IRDs and patients with *ARL2BP*-related retinal dystrophies are no exception. A relative early loss of BCVA with HM (or even LP) in the fourth decade of life as well as BCVA of 20/40 up to the sixth decade of life have both been reported. At the same time, marked macular atrophy, posterior subcapsular cataracts and epiretinal membrane could be present [11].

Although patients with specific IRDs share some typical phenotypic traits, these kinds of diseases do not always occur and advance in a similar way among members of the same family.

The clinical history of 3 affected sibs from a consanguineous Arab family which tested positive for a homozygous c.101-1G > C transversion at the splice acceptor site of intron 2 of the *ARL2BP* gene is an example [1]. The absence of a normally spliced transcript determined the symptoms of retinitis pigmentosa (NB and VFC) at their 20s in all three patients as well as macular atrophy with epiretinal membrane and scotopic and photopic ERG responses not differentiable from background noise. However, thoracic and abdominal situs inversus was complete in the older sister and brother, whereas the younger brother had normal positioning of the thoracic and abdominal organs.

Another splice site variant in intron 3 (c.207 + 1G > T) was reported in two Moroccan sisters, daughters of consanguineous parents and affected by rod-cone dystrophy. In these patients, chest radiographs did not show situs inversus but one of them, at the age of 36, had 20/200 BCVA in the RE and counting fingers in the LE with a visual field inferior to 10 degrees while her 27 years old sister, showed 20/25 BCVA in both eyes and visual field less than 30 degrees. Fundus retinoscopy showed bone spicule pigmentation, attenuation of retinal vessels and pale optic disks which in the older sister were accompanied by macular atrophy [5].

Few studies document the correlations between *ARL2BP* gene variants and their respective systemic and ophthalmological signs. Given the role of the *ARL2BP* protein in the ciliary complex, it is perhaps not surprising that infertility is confirmed to occur in male patients, as already demonstrated in mouse models [12, 13] and in two male patients, one affected by RP and asthenozoospermia without situs inversus [12] and the second with RP, situs inversus totalis, and oligozoospermia [6].

Live images concerning sperm motility in murine *ARL2BP* KO models demonstrated that sperm were immotile while testis size and weight were comparable between WT and KO. Despite a normal spermatogenesis in KO animals, sperm release into the lumen was impaired, with a smaller lumen area, absent or spiraled sperm tails and other gross morphological defects.

With these inabilities, the number of sperm in the epididymis of these mice was drastically reduced and all had gross morphological defects. In consideration of these results, it became clear that *ARL2BP* did not interrupt the initiation of microtubular axoneme growth but blocked its maturation [12]. These outcomes are largely comparable to those encountered in our patient. His semen analysis had watery appearance, smaller volume and increased viscosity. The sperm count was significantly lower than normal (5.000.000/ml instead of ≥ 50.000.000/ml) with an absent motility two hours after release. Sperm morphology was atypical since spermatozoa had amorphous heads, microcephaly and 100% docked tails.

Since IFT defects can cause situs inversus, retinal degeneration and polycystic kidney disease, it would be interesting to check if women affected by this syndrome also have infertility and to confirm if sperm abnormality are common in affected males.

In our patient a renal microcyst in the right kidney and an oval structure of approximately 7 cm characterized by a cluster of small cysts on the left side, posterior to the bladder, were encountered.

This is the first time that such a feature is documented in an *ARL2BP* patient. However, although it is novel, it is explainable by the impact on cilia and unlikely to be a co-incident condition since renal agenesis and effects on male fertility are reported in other ciliopathies like BBS [14, 15].

In addition, we do not know whether the apparent left renal agenesis is associated with the presence of cryptorchidism of the left testicle, which has also never been described so far.

Another finding that we are documenting for the first time in this patient is his positive result to anti-retinal autoantibodies against 30-kDa, 35-kDa and 44-kDa proteins. In view of the result of the genetic analysis, we believe that positivity to anti-retinal autoantibodies is an epiphenomenon of retinitis pigmentosa caused by biallelic variant in the *ARL2BP* gene. The development of anterior uveitis could also be related to the presence of autoantibodies, but we cannot establish with certainty, because the immunological test was not performed before the acute event.

Although this patient is suffering from severe and advanced retinal degeneration, he continues to have a functional residual vision at 63 years, which proves the phenotypic variability of the IRDs.

The collection of clinical data from previous ophthalmological evaluations allowed us to analyze the natural history of this rare retinal dystrophy over time.

Based on a recent literature review, Fiorentino et al. briefly described the progression of this disease over time in two patients lasting 7 and 9 years [4] and Moye et al. reported other two clinical stories of *ARL2BP* patients [12].

The features of the mentioned cases are presented in Tables 2 and 3 in order to provide a more concise summary of the literature and the presence/absence of known findings.

**Table 2:** General features of all patients with ARL2BP-related retinal dystrophies described in the scientific literature

Nr	Sex	Ethnicity	Pathogenic variant	Status	General features					Reference
					Situs inversus	Infertility	Kidney disease	Cryptorchidism	Hearth defects	
1	F	Arab-Muslim	c.101-1G > C	HOM	YES (complete)	NR	NR	NO	NO	Davidson AE et al. [1]
2	M	Arab-Muslim	c.101-1G > C	HOM	YES (complete)	NR	NR	NR	NO	Davidson AE et al. [1]
3	M	Arab-Muslim	c.101-1G > C	HOM	NO	NR	NR	NR	NO	Davidson AE et al. [1]
4	M	European	c.134T > G; p.(Met45Arg)	HOM	NR	NR	NR	NR	NO	Davidson AE et al. [1]
5	F	White	c.390 + 5G > A	HOM	NR	NR	NO	NO	NO	Fiorentino A et al. [4]
6	M	North African	c.207 + 1G > A	HOM	NI	NR	NR	NR	NO	Fiorentino A et al. [4]
7	F	North African (Moroccan)	c.207 + 1G > A	HOM	NO	NR	NR	NO	NO	Audo I et al. [5]
8	F	North African (Moroccan)	c.207 + 1G > A	HOM	NO	NR	NR	NO	NO	Audo I et al. [5]
9	M	European (Portugal)	c.207 + 1G > A	HOM	NR	YES	NR	NR	YES	Moye AR et al. [12]
10	F	European (Portugal)	c.33_36delGTCT; p.(Phe13Profs*15)	HOM	NR	NR	NR	NR	NO	Moye AR et al. [12]
11	M	Asian (Chinese)	c.22_23delAG; p.(Ser8Leufs*10)	HOM	YES (complete)	YES	NR	MD	NR	Zhu T et al. [6]
12	M	European	c.294-1G > C	HOM	YES	YES	YES	YES	YES (mitral valve prolapse without regurgitation)	Placidi G et al.

*Legend* HET = Heterozygous; HOM = Homozygous; F = Female; M = Male; MD = Missing data; NI = Not investigated; NR = Not reported

**Table 3:** Ophthalmic features of all patients with ARL2BP-related retinal dystrophies described in the scientific literature

Nr	Sex	Ethnicity	Pathogenic variant	Status	Age of onset	First symptoms	Ophthalmic features				Reference
							Cataract	Epiretinal membrane	Bone-spicule-like pigmentation	Macular atrophy	
1	F	Arab-Muslim	c.101-1G > C	HOM	**~ 20**	NB and VFI	YES (posterior subcapsular)	YES	YES (mild-to-moderate)	YES	Davidson AE et al. [1]
2	M	Arab-Muslim	c.101-1G > C	HOM	**~ 20**	NB and VFI	NR	YES	NR	YES	Davidson AE et al. [1]
3	M	Arab-Muslim	c.101-1G > C	HOM	**~ 20**	NB and VFI	NR	YES	NR	YES	Davidson AE et al. [1]
4	M	European	c.134T > G; p.(Met45Arg)	HOM	**~ 20**	NB	NR	NO	YES (sparse)	NO	Davidson AE et al. [1]
5	F	White	c.390 + 5G > A	HOM	35	SDA and PH	NR	NO	YES (mainly in the nasal retina)	NO but perimacular rings of increased signal in FAF	Fiorentino A et al. [4]
6	M	North African	c.207 + 1G > A	HOM	36	NB	NR	NO	YES (widespread)	YES	Fiorentino A et al. [4]
7	F	North African (Moroccan)	c.207 + 1G > A	HOM	MD	MD	MD	NO	MD	MD	Audo I et al. [5]
8	F	North African (Moroccan)	c.207 + 1G > A	HOM	MD	MD	MD	YES	MD	MD	Audo I et al. [5]
9	M	European (Portuguese)	c.207 + 1G > A	HOM	26	NB and PHT	YES (moderate)	NO (mild ILM thickening)	YES (scarce along the superior vascular arcad)	YES	Moye AR et al. [12]
10	F	European (Portuguese)	c.33_36delGTCT; p.(Phe13Profs*15)	HOM	11	PH	YES (pseudophakic)	NO	YES (scarce, adjacent to the optic nerve head and periphery)	YES (central atrophic lesion)	Moye AR et al. [12]
11	M	Asian (Chinese)	c.22_23delAG; p.(Ser8Leufs*10)	HOM	MD	MD	MD	MD	MD	MD	Zhu T et al. [6]
12	M	European	c.294-1G > C	HOM	**~ 20**	PH and PHT	YES (mild lens sclerosis)	NO	YES (rare, scattered in mid-periphery)	YES	Placidi G et al.

*Legend* HET = Heterozygous; HOM = Homozygous; F = Female; ILM = Internal limiting membrane; M = Male; MD = Missing data; NB = Night blindness; NI = Not investigated; NR = Not reported; PH = Photophobia; PHT = Photopsia; SDA = Slow dark adapatation; VFI = Visual field impairment

In our patient we reconstructed 30 years of disease history. The graphs shown in Fig. 4 highlight a clear progression of the retinal degenerative process but data collected over the last 15 years indicate a slower rate of progression at the end-stage retinal disease when compared with the first 15.

In addition, about halfway along his clinical route, an anterior uveitis in both eyes occurred and the patient underwent periocular injections of steroids. This event certainly had a decisive impact on the evolution of his disease and we cannot determine how his retinal dystrophy would have progressed if it had not happened.

To the best of our current knowledge, FST results have never been reported on patients with IRD due to biallelic variants in *ARL2BP* gene. The main outcome of this examination has been that isolated rod-function response was almost equal to cone-mediated response in both eyes.

Although the retinal sensitivity of this patient was abnormally reduced in both eyes, responses were reliable with nearly halved values in RE compared to LE. These results, together with the ERGs, indicate that this retinal dystrophy has a mixed component and affects rods on broadly the same rate as cones. It is no coincidence that photophobia was the first symptom and only ten years later was nyctalopia noticed as well as the patient reported better visual performances in lower lighting environments.

*ARL2BP* patients currently described in the medical literature are still extremely rare. Therefore, the phenotypic manifestations of this syndrome have not yet been fully addressed. As in other IRDs and pleiotropic genetic disorders, patients show a variable expressivity of their disease and a wide range of clinical differences can be observed both within and between families.

Furthermore, other factors that are not fully understood, such as mutational load [16, 17], altered immunoregulation [18] and gender effects [19] should be considered. In conclusion, the novel splice site variant we mentioned broadens the genotypic spectrum of the previously reported variants while the novel clinical findings in this patient extend the phenotypic spectrum of *ARL2BP*-associated ciliopathies.

This extensive analysis provides long-term outcomes that may be possible in *ARL2BP* and constitutes an added value since it could facilitate clinical diagnosis and patient management. In this regard, given the multi-organ involvement, it should be stressed that the implication of this genetic condition require an integrated multidisciplinary team harbouring different specialists. Not only ophthalmologists and geneticists are needed but also internists, nephrologists, andrologists/urologists and orthoptists with experience in visual rehabilitation.

In addition, this case report shows the progression of this specific kind of novel variant.

As previously suggested, IRDs due to *ARL2BP* variants are not always associated with situs inversus and male infertility was reported only in three cases, including ours. Given the presence of renal cysts, which can occur in other ciliopathies, a differential diagnosis with SLS, BBS and JS should be considered, especially when the clinical phenotype does not exhibit all the specific manifestations.

Fortunately, this patient did not show features of other ciliopathies in the differential and the absence of diagnostic criteria for BBS or JS facilitated the molecular genetic classification.

## Data Availability

The datasets used and/or analysed during the current study are available from the corresponding author on reasonable request.

## Abbreviations

ARL2BP: ADP-ribosylation factor-like 2 binding protein
BBS: Bardet Biedl Syndrome
BCVA: Best corrected visual acuity
ERG: Electroretinogram
FAF: Fundus autofluorescence
ERG: Electroretinogram
FST: Full-field stimulus threshold testing
HM: Hand motion
LP: Light perception
IFT: Intraflagellar transport
IRD: Inherited retinal dystrophy
ISCEV: International Society for Clinical Electrophysiology of Vision
JS: Joubert Syndrome
KO: Knockout
LE: Left eye
NB: Night blindness
NGS: Next generation sequencing OS Outer segment
RE: Right eye
RNFL: Retinal Nerve Fiber Layer
RP: Retinitis pigmentosa
RPE: Retinal pigment epithelium
SLS: Senior-Loken Syndrome
VEP: Visual evoked potential
VFC: Visual field constriction
WT: Wild-type
